# Pivoting the Provision of Smoking Cessation Education in a Virtual Clinical World: The Princess Margaret Cancer Centre Experience

**DOI:** 10.3390/curroncol28060449

**Published:** 2021-12-15

**Authors:** Naa Kwarley Quartey, Janet Papadakos, Ben Umakanthan, Meredith Elana Giuliani

**Affiliations:** 1 Cancer Education Program, Princess Margaret Cancer Centre, Toronto, ON M5G 2C4, Canada; naakwarley.quartey@uhnresearch.ca (N.K.Q.); janet.papadakos@uhnresearch.ca (J.P.); ben.umakanthan@uhnresearch.ca (B.U.); 2 Institute of Health Policy, Management & Evaluation, University of Toronto, Toronto, ON M5T 3M6, Canada; 3 Patient Education, Ontario Health (Cancer Care Ontario), Toronto, ON M5G 2L3, Canada; 4 Radiation Medicine Program, Princess Margaret Cancer Centre, Toronto, ON M5G 2M9, Canada; 5 Department of Radiation Oncology, University of Toronto, Toronto, ON M5T 1P5, Canada

**Keywords:** smoking cessation, patient education, health professions education, virtual care

## Abstract

Continued smoking after a cancer diagnosis may be attributed to misbeliefs by both patients and healthcare providers on the value and benefit of quitting smoking on treatment outcomes. The perceived myths and misconceptions about the relationship between smoking and cancer may be readily dispelled with the provision of practical and pertinent education. However, busy clinics as well as the rapid move to virtual care due to the COVID-19 pandemic present several challenges with the provision of smoking cessation education. Here, we describe how the Princess Margaret Cancer Centre implemented innovative solutions to improve the delivery of education during the COVID-19 pandemic to better support patients and healthcare providers.

## 1. Introduction

Quitting smoking after a cancer diagnosis has been shown to improve treatment response [1], increase cancer-specific and overall survival outcomes [2], reduce the risk of cancer recurrence [3] and prevent secondary cancers [4]. Despite the well-documented benefits of smoking cessation, many patients continue to use commercial tobacco products throughout cancer treatment [5,6,7]. To a substantial degree, continued smoking is attributed to misbeliefs among patients and healthcare providers about the value of quitting after a cancer diagnosis. These misbeliefs, including, “it is too late to quit smoking after a diagnosis”, and that “discussing smoking cessation is not appropriate when a patient is newly diagnosed” [5], are some of the reported misconceptions that result in patients continued use of tobacco through treatment and beyond. Previous studies have demonstrated that many cancer patients are not aware of the continued harms of smoking on their treatment outcomes [6,8]. These misconceptions and misbeliefs about the relationship between smoking and cancer may be dispelled with the systematic provision of practical education.

Cancer centres play a critical role in reducing the incidence of smoking among newly diagnosed patients, and effective strategies and supports should be in place to ensure all patients, particularly those with low or limited health literacy, understand the harms of continued smoking after a diagnosis of cancer. Patients should be made aware of the harms of continued smoking and be provided with resources to support smoking cessation. It is the responsibility of cancer centres to meet the needs of patients by providing them with access to actionable and understandable health information [9] and to direct them to services for continued counselling. Additionally, healthcare providers must be supported with education and training to be better equipped to educate patients within their care.

Comprehensive tobacco cessation programs, such as the one implemented at the Princess Margaret Cancer Centre, consist of three key elements that provide numerous opportunities for the dissemination of information and education to patients: (1) ASK: ensure all newly diagnosed cancer patients are asked about their smoking status within 6 months of their diagnosis, (2) ADVISE: provide patients with tailored educational information on the benefits of quitting smoking before and during treatment and (3) ACT: refer patients to internal or external cessation services for support [10,11].

This paper describes the landscape of educational requirements for patients with cancer who smoke, and reflects on the challenges, opportunities, and innovative solutions for the provision of education, many of which were implemented because of the COVID-19 pandemic.

## 2. Landscape of Educational Requirements for Smoking Cessation among Cancer Patients

The provision of education is a critical aspect of tobacco cessation [12]. To better understand the information that patients want to know, we conducted an assessment to explore their educational needs related to smoking across five domains: (1) General Information and Support, (2) Smoking Health and Disease, (3) Relationships, (4) Testimonials and (5) Interventions [13]. Participants were presented with informational need items and asked to rate the importance and the amount of information they would like to receive on each item. Additionally, for each domain, they ranked their top three modality preferences for receiving this information. Our needs assessment revealed that patients who smoke want a significant amount of information about smoking and cancer, namely in the domains of General Information and Support as well as Smoking, Health and Disease [13]. It further showed that patients want information about the harms of smoking on their cancer treatment, the positive impact of quitting on their response to treatment and an understanding of the reasons why some patients continue to smoke even when made aware of the subsequent risks [13]. Patients indicated that they preferred to receive this information in pamphlet form [13]. Although access to digital health information has proliferated in recent years, the preference for print materials is more common among individuals with low or limited health literacy [14]. Low health literacy is associated with less knowledge about smoking health risks and lower risk perceptions. In addition, low health literacy may be linked to a patient’s inability to quit smoking [15,16]. Patients with low or limited health literacy and those with low socioeconomic status are at greatest risk of smoking [17,18] and are subsequently at greatest risk of a poorer treatment response [19].

The provision of practical and pertinent information that adheres to best practices in health literacy has been shown to increase disease self-management among patients diagnosed with cancer [20,21]. Healthcare providers must be able to provide this information to patients in a timely manner [13]. However, in the context of busy clinical environments, the process for disseminating this information needs to be easy and seamless without interruption to clinical workflow. In appreciation of this need, we adopted a multipronged approach to delivering education to patients as well as supporting healthcare providers who counsel patients on smoking cessation. The cancer centre offers patients a range of educational interventions to aid with their efforts to reduce or quit smoking. This includes traditional educational resources, such as pamphlets, available to patients in the library as well as in clinic waiting areas. Additionally, posters with tailored, treatment-specific messaging are distributed throughout the cancer centre and are available on digital screens, to ensure patients receive repeated reminders about smoking cessation in and out of the clinic setting. This strategy reinforces the importance of smoking cessation at any point along the cancer journey. While the newly diagnosed stage presents an optimal teachable moment to discuss smoking cessation strategies, patients may be overwhelmed with the amount of information given to them at the time of diagnosis. Patients surveyed in our needs assessment did not have a preference regarding the timing of receiving information about smoking cessation [13], thus, reminders throughout the cancer centre may provide optimal opportunities to reinforce the importance of quitting smoking through brief educational messaging. All education materials created within the cancer centre are developed according to best practices in health literacy. That is, ensuring they are understandable and actionable [22] and written at appropriate reading grade levels [23,24] so that they may be better understood by patients with low health literacy or limited English proficiency.

Our multipronged strategy demonstrates that a one-sized fits all approach to providing education to patients is not effective. Rather, education needs to be available in multiple modalities and in multiple languages, to meet the needs of the diverse patient populations we serve. To have a lasting impact, education must also be reinforced at various points along the cancer trajectory.

To effectively educate patients on the benefits of quitting smoking, it is critical that healthcare providers also receive training to be better equipped to provide patients with the information they need to help them quit. Programs, such as the Training Enhancement in Applied Counselling and Health (TEACH), offer evidence-based, clinical information on the importance of tobacco cessation [25]. The TEACH program consists of over 40 h of training to prepare healthcare providers for intensive counselling to support patients in their efforts to quit smoking [25]. While this comprehensive course prepares healthcare providers to counsel and educate patients on the harms of commercial tobacco use, healthcare providers in busy clinic environments are likely unable to participate in these courses due to time constraints. Rather, the use of microeducational interventions, such as short informational videos, can be an effective strategy in the dissemination and retention of information among clinicians and can improve healthcare providers’ confidence in discussing approaches to smoking cessation [26]. The implementation of microcourses or other short educational videos that are actionable and reinforce key messages for patients on the benefits of quitting smoking after a cancer diagnosis, may be a more sustainable solution to educating larger numbers of clinicians and may be more readily adopted in the clinic setting. Improved educational strategies for healthcare providers may further encourage patients to seek referrals to internal or external services for ongoing support and counselling.

## 3. Challenges and Opportunities Presented by the COVID-19 Pandemic

Following provincial mandates, all newly diagnosed patients at the cancer centre should be assessed for smoking status by completing a self-reported outcome measure, CEASE [10]. Prior to the COVID-19 pandemic, assessments were completed either on paper or on iPads located in outpatient ambulatory clinics. Assessments completed on iPads allowed for comprehensive screening and subsequent automatic referrals through a process intended to reduce the additional work required by clinic staff [27], while paper forms were manually entered into a central database for monthly reporting. Patients can complete CEASE assessments in English or five other languages. Unfortunately, the number of newly diagnosed cancer patients screened for smoking status each month falls short of provincial targets due to several reasons. Discussions with clinic staff revealed that high staff turnover as well as lack of awareness by staff on the importance of screening patients for smoking status were two factors that negatively impacted the success of meeting provincial screening targets. Further, the multidisciplinary nature of cancer treatment may result in some new patients seeing members of their healthcare team at institutions outside the cancer centre. These hospital institutions do not have formal processes for screening newly diagnosed cancer patients for smoking status, as this is not part of their existing clinical workflow.

Given current in-person health service reductions due to the COVID-19 pandemic, virtual clinics have become essential for meeting the needs of patients and families [28,29]. The rapid move to virtual care resulted in the cancer centre smoking cessation program facing several challenges; however, each challenge presented opportunities to improve the program for both patients and healthcare providers. Princess Margaret at Home (PATH) and the Digital Education Prescription (DEP), are two solutions implemented during the COVID-19 pandemic to better support patients and healthcare providers at the cancer centre.

PATH was implemented to increase patient access to and improve the convenience of completing assessments, including CEASE, online up to 48 h before clinic visits. Results of assessments are integrated with the Virtual Care Management System (VCMS) [30], allowing healthcare providers to easily view reports and subsequently counsel patients on tobacco cessation strategies. PATH still includes the automation of CEASE assessments and referrals, while permitting patients to conveniently complete assessments prior to their clinic visit. This process allows for staff to spend less time administering assessments and more time reviewing them and educating patients on the benefits of smoking cessation on their cancer and treatment. Unfortunately, once referrals are made to external or internal sources for support, there is no formal follow up (e.g., quit statistics) on these patients.

The DEP is a secure online tool that allows healthcare providers to email educational resources to patients and their families [31]. The DEP exists within an educational infrastructure at the cancer centre and is a repository of over 1900 of multimodal patient education resources available in 24 languages. The repository includes links to a variety of resources, including pamphlets, videos, eLearning classes and apps created in the cancer centre or by external organizations. The information within the DEP remains current and patients receive up-to-date information, as it is integrated with an existing patient education Content Management System (CMS). The CMS is managed internally by the organizational patient education department and this oversight ensures that, following best practice guidelines, resources created within the institution are reviewed every 3 years by subject matter experts for safety and accuracy. External resources that are added to the DEP are kept current using a tool that validates links to ensure they are active.

Working closely with healthcare providers across the cancer centre, individual clinic profiles and special topics were curated with tailored resources to meet the unique needs of the patient population within each clinic. One special topic includes a dedicated profile containing all available patient education on smoking cessation. Healthcare providers can select the appropriate resources from these lists and easily add them to an email sent from a generic account. To improve ease of access to the DEP, a link to the prescription is integrated with VCMS to allow for a seamless experience for clinicians to prescribe education along with other tests, procedures, and referrals. While access to the DEP has not replaced the provision of traditional paper resources given to patients during in person visits, the DEP has become essential at a time when many more patients are seen through virtual care.

The introduction of the DEP has improved the ease with which staff provide patients with accurate and up-to-date information. This increased trust in the provision of information from healthcare providers further underscores the importance of education provision by staff as patients have a greater trust in information given to them from their healthcare providers compared to information that patients source themselves [32].

## 4. Conclusions

Access to high-quality educational resources is essential for meeting the informational needs of patients newly diagnosed with cancer who smoke. The advent of digital solutions has facilitated the provision of multimodal educational resources, particularly at a time when most patients are seen through virtual care, and these digital solutions have greatly impacted the provision of the cancer centre smoking cessation program. Digital educational solutions that follow best practices in health literacy and patient education have allowed healthcare providers to offer a range of educational interventions to meet the needs of our diverse patient population and encourage sharing of information.
